# Design of a time-delay-compensated monochromator for the ARPES endstation at S^3^FEL

**DOI:** 10.1107/S1600577525002139

**Published:** 2025-04-02

**Authors:** Zhenjiang Xing, Chuan Yang, Qinming Li, Kai Hu, Ye Zhu, Chen Wu, Chenggong Zhang, Weiqing Zhang

**Affiliations:** a Institute of Advanced Science Facilities, Shenzhen518107, China; b State Key Laboratory of Molecular Reaction Dynamics, Dalian Institute of Chemical Physics Chinese Academy of Sciences Dalian116023 China; Bhabha Atomic Research Centre, India

**Keywords:** free-electron laser, X-ray beamline design, time-delay-compensated monochromator, Fourier-transform limited pulse, ultrashort pulse propagation

## Abstract

The preliminary design and expected performance for the Angle-Resolved Photoemission Spectroscopy (ARPES) branchline at Shenzhen Superconducting Soft X-ray Free Electron Laser (S^3^FEL) is presented. A time-delay-compensated monochromator (TDCM) in symmetric layout has been designed for spectral selection and pulse duration preservation. The TDCM is optimized using the six-dimensional **K**-matrix method and a start-to-end simulation of the beamline system using Fourier optics was performed. Numerical estimations indicate that the TDCM can achieve a time–bandwidth product approaching the Fourier-transform limit.

## Abstract 2

This work presents the design and expected performance of an Angle-Resolved Photoemission Spectroscopy (ARPES) branchline at the Shenzhen Superconducting Soft X-ray Free Electron Laser (S^3^FEL). The branchline employs a symmetric time-delay-compensated monochromator (TDCM) for spectral selection and pulse duration preservation. Numerical optimization using the six-dimensional **K**-matrix method and Fourier optics simulation shows that the TDCM can achieve a time–bandwidth product approaching the Fourier-transform limit.

## 1. Introduction

The remarkable advancement of the free-electron laser (FEL) has provided a high-performance light source characterized by high spatial coherence, ultra-short pulse duration and extreme brightness (Ackermann *et al.*, 2007 ▸; Emma *et al.*, 2010 ▸; Ishikawa *et al.*, 2012 ▸; Allaria *et al.*, 2012 ▸; Kang *et al.*, 2017 ▸; Milne *et al.*, 2017 ▸; Decking *et al.*, 2020 ▸; Zhao *et al.*, 2019 ▸; Zhang *et al.*, 2023 ▸). These FEL facilities have been extensively applied to basic studies in atomic and molecular physics, biological materials and condensed matter physics (Li *et al.*, 2024 ▸; Kinschel *et al.*, 2020 ▸; Först *et al.*, 2015 ▸). Despite the outstanding performance of FELs, challenges still exist in realizing time-resolved spectroscopy studies, such as time-resolved and angle-resolved photoemission spectroscopy (tr-ARPES) (Liu, 2023 ▸), which requires the source to have high temporal resolution (typically less than 100 fs) and high energy resolution (typically less than 100 meV) to precisely detect ultrafast electron dynamics and nonequilibrium electronic distribution in materials. Although different experiments may employ various compromises between temporal and energy resolution, the time–bandwidth product of pulses generally approaches the Fourier-transform limit of Gaussian pulses (less than three times the Fourier-transform limit for most experiments) (Gauthier *et al.*, 2020 ▸). However, the current operation models of FELs, such as self-amplified spontaneous emission (SASE) and echo-enabled harmonic generation (EEHG), struggle to produce FEL pulses with such desired performance (Düsterer *et al.*, 2011 ▸; Allaria *et al.*, 2012 ▸; Zhao *et al.*, 2017 ▸; Milne *et al.*, 2017 ▸; Hemsing *et al.*, 2017 ▸; Maroju *et al.*, 2020 ▸; Liu *et al.*, 2023 ▸). Therefore, it is necessary to manipulate FEL pulses using optical devices to meet the experimental conditions. A typical beamline for tr-ARPES requires an optimized monochromator to perform spectral selection through a slit while preserving the ultrashort pulse duration or inducing a tolerable pulse stretching.

Monochromatization in the extreme-ultraviolet and soft X-ray ranges typically utilizes diffractive elements, such as gratings, which can result in stretching of the pulse duration (tens or even hundreds of femtoseconds) due to pulse front tilt (Poletto & Frassetto, 2010 ▸). There are some strategies to minimize the pulse stretching in a single-grating monochromator through the optimization of parameters, such as using the gratings in the off-plane mount or clipping the beam to limit the number of illuminated grooves (Frassetto *et al.*, 2011 ▸; Nicolas & Cocco, 2022 ▸). However, these methods do not fully meet the stringent requirements of the ideal time–bandwidth product as described by the Fourier-transform limit, which represents the minimum achievable product of pulse duration and spectral bandwidth for a given light source. The design of two consecutive gratings with opposite orders, also called the time-delay-compensated monochromator (TDCM), has been proposed to reduce pulse stretching induced by pulse front tilt (Villoresi, 1999 ▸). In such a configuration, the first grating is used for spectral selection on an intermediate slit, while the second grating compensates for the pulse front tilt introduced by the angular dispersion of the first one. The gratings should be able to achieve both dispersion and focusing. Some studies (Ito *et al.*, 2010 ▸; Poletto *et al.*, 2011 ▸; Igarashi *et al.*, 2012 ▸; Poletto *et al.*, 2018 ▸) have already constructed similar time-delay-compensated monochromators based on a toroidal grating pair or a variable-line-spacing (VLS) grating pair. These works all conducted simulations using geometric optics or ray tracing models. These models can roughly evaluate the impact of pulse front tilt and group delay on the pulse duration, but they cannot precisely calculate the specific effects of diffraction on pulse shape and temporal response, such as diffraction induced by the intermediate slit, which will be the primary contributors to pulse stretching on high-resolution monochromators.

In this paper, we present the preliminary design of a TDCM for the ARPES branchline of the FEL-4 beamline system at Shenzhen Superconducting Soft X-ray Free Electron Laser (S^3^FEL), which utilizes a pair of VLS gratings with opposite orders for monochromatization and time delay compensation. In the design, we first conduct a theoretical analysis of the monochromator using a 6 × 6 matrix (Kostenbauder matrix, or **K** matrix) (Kostenbauder, 1990 ▸; Marcus, 2016 ▸; Hu *et al.*, 2023*b* ▸) and then we carry out a start-to-end simulation of the beamline based on the pulse propagation method of Fourier optics (Hu *et al.*, 2023*a* ▸; Zhu *et al.*, 2024 ▸). The influence of various factors on pulse shape and temporal response is investigated, which indicate the potential of the TDCM to achieve a time–bandwidth product approaching the Fourier-transform limit. Before discussing the details of the TDCM design, we will introduce the evaluation methods utilized in this paper.

## 2. Pulse propagation calculation method

In this section, we provide a brief overview of two different methods for the propagation of ultra-short pulses. The first one is based on the six-dimensional (6-D) **K** matrix, and the second one utilizes the pulse propagation method based on Fourier optics.

### 2.1. Pulse propagation method based on the 6-D **K** matrix

As a simple and elegant method for describing spatio-temporal couplings, the **K**-matrix method is a powerful tool for calculating the propagation of ultra-short pulses in dispersive systems. This method geometrically and linearly describes the spatio-temporal effects and dispersion properties induced by the system. The **K** matrix of an optical system describes the propagation characteristics, allowing for an independent description of spatio-temporal properties inherent in an optical element or the entire beamline without the specific knowledge of incident light field. Additionally, this method can also be adapted to describe the propagation properties of Gaussian pulses through analytical expressions. Detailed descriptions of the **K** matrix have been given by Hu *et al.* (2023*b* ▸) and Kostenbauder (1990 ▸), with only a brief overview provided here to understand its fundamental principles.

**Figure 1:**
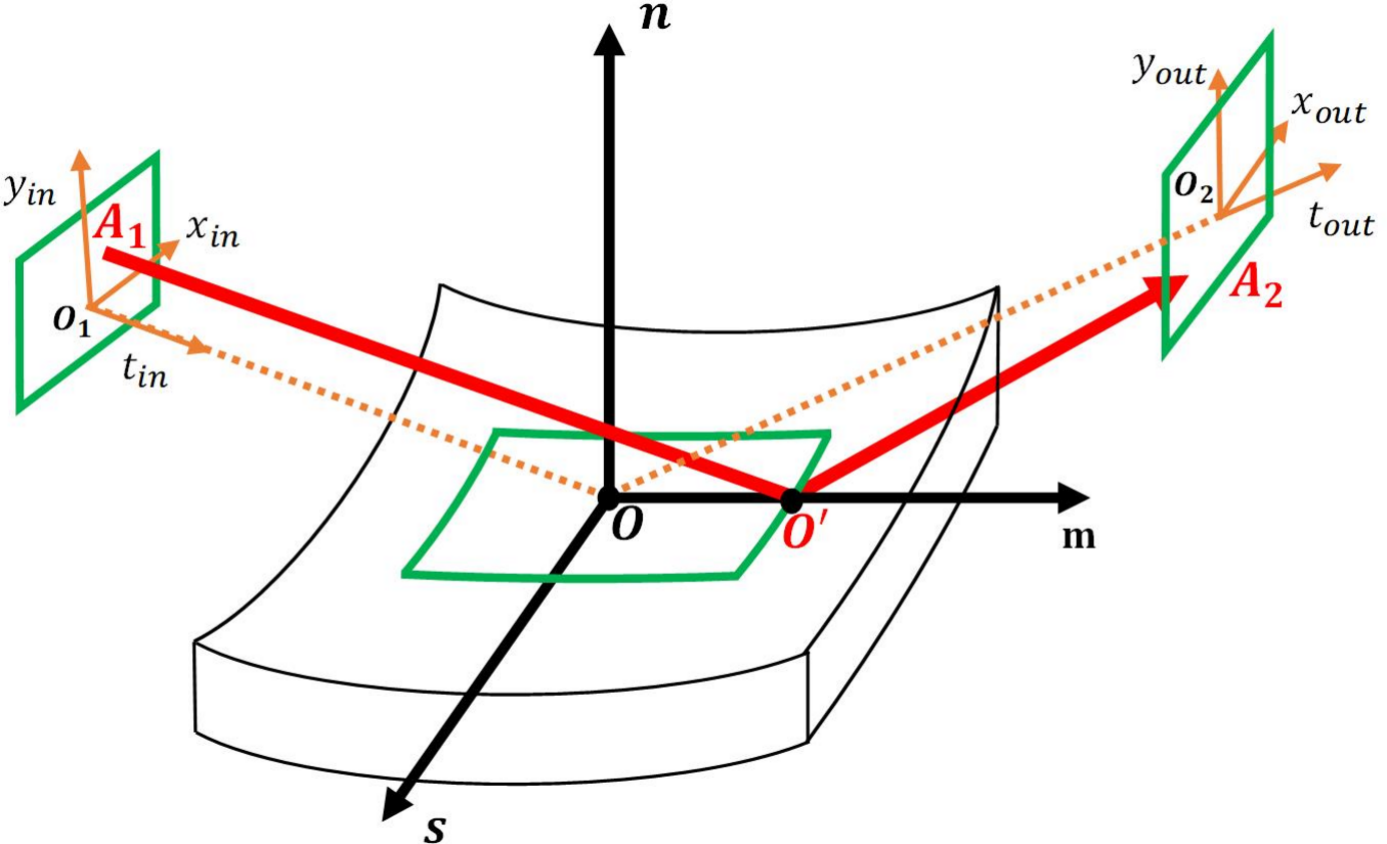
Schematic illustration of the coordinate definition at the 6-D **K** matrix.

The optical axis is defined as the path of the reference ray with a given central frequency *f*_0_ through the optical system. The transverse coordinate plane (*x*, *y*) is constructed orthogonal to the optical axis, and the reference ray at the optical axis is located at the origin of the transverse coordinate plane. For example, the coordinate where a pulse ray passes through an upward optical element is shown in Fig. 1 ▸.

The X-ray pulse is described by a 6-D phase space vector **V** = (*x*, θ_*x*_, *y*, θ_*y*_, *t*, ν)^T^, which is defined as the deviation of the studied ray relative to the reference ray. Here, *x* and *y* represent the transverse deviation of a specific ray relative to the reference ray, θ_*x*_ and θ_*y*_ describe the angular deviation relative to the optical axis, and *t* and ν represent the time deviation and frequency deviation, respectively, relative to reference time and the central frequency *f*_0_, and ν = *f* − *f*_0_.

The relation between the groove density *n* and the grating coordinate along the meridian direction *w* can be expressed as *n* = 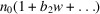, where *n*_0_ is the central groove density and *b*_2_ is the VLS parameter (Harada & Kita, 1980 ▸; Hettrick & Underwood, 1986 ▸). Based on the first-order spatio-temporal response properties, the 6-D **K** matrix for the upward toroidal VLS grating can be expressed as (Hu *et al.*, 2023*b* ▸) 
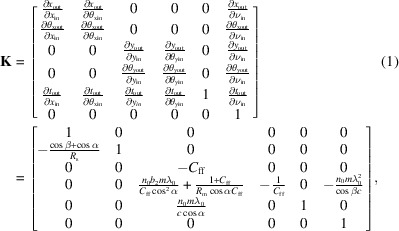
where λ_0_ is the central wavelength and *c* is the speed of light in a vacuum. The grooves of the VLS grating are perpendicular to the meridian direction, with a central groove density *n*_0_ and VLS parameter *b*_2_. α and β are the incident and diffracted angles, respectively, with respect to the normal. *C*_ff_ = 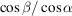 is the fixed-focus constant of the grating. *R*_s_ and *R*_m_ represent the radius of curvature in the sagittal and meridian dimensions, respectively. 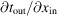 and 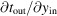 represent the pulse front tilt for each spatial direction. 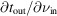 represents the system on-axis dispersion related to group delay dispersion (GDD).

This matrix can also be reduced to describe toroidal VLS gratings, cylindrical VLS gratings, planar VLS gratings, toroidal gratings, cylindrical gratings, planar gratings, toroidal mirrors, cylindrical mirrors and planar mirrors.

### 2.2. Pulse propagation method based on Fourier optics

The **K**-matrix method cannot describe the complex diffraction effects of the optical system, since it is based on geometric optics. The simulation tool *FURION* (*Fourier optics-based Ultrashort x-Ray pulse propagatION* tool) (Zhu *et al.*, 2024 ▸) is a start-to-end optical computational software program, capable of simulating 3-D pulse propagation in X-ray FEL (XFEL) beamline systems for both non-dispersive and dispersive systems. The pulse optical field is stored in a 3-D complex matrix with sampling points of *N*_*x*_ × *N*_*y*_ × *N*_*t*_, where *N*_*x*_ and *N*_*y*_ denote the number of sampling points in the transverse coordinates (*x* and *y*), and *N*_*t*_ represents the number of sampling points in the longitudinal coordinate (*t*).

The 3-D optical field propagation through free space with distance *d* can be expressed as (Zhu *et al.*, 2024 ▸) 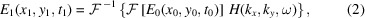
where *E*_0_(*x*_0_, *y*_0_, *t*_0_) is the initial 3-D optical field, and 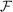 and 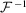 are Fourier transform and inverse Fourier transform, respectively. *H*(*k*_*x*_, *k*_*y*_, ω) is the transfer function of free space propagation, which can be written as 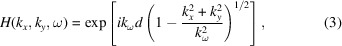
where *k*_ω_ = ω/*c*. *k*_*x*_ and *k*_*y*_ are the projection components of the wavevector *k*_ω_ in the *x* and *y* dimensions, respectively.

According to the **K**-matrix method, while the pulse travels through a dispersive system, such as a toroidal VLS grating, there are three different effects: transverse scaling, focusing and angular dispersion.

Transverse scaling is caused by the fixed-focus constant *C*_ff_ for a toroidal VLS grating, which can be written as 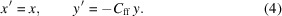
Angular dispersion modulation and focusing modulation of a toroidal VLS grating can be expressed as 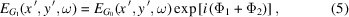
where Φ_1_ and Φ_2_ represent the phase terms of focusing modulation and angular dispersion modulation, which can be described as 
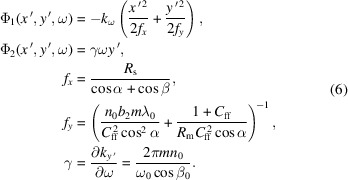
By meticulously adjusting the parameters *C*_ff_, *n*_0_, *b*_2_, *R*_s_ and *R*_m_, we can calculate the propagation of 3-D XFEL pulses through various optical components, including VLS gratings, equal-line-spacing gratings and reflective mirrors.

## 3. Monochromator design

In this section, we utilize the **K** matrix to conduct a theoretical analysis of the monochromator consisting of two VLS gratings for the ARPES branchline of the FEL-4 beamline system at S^3^FEL, and discuss the impact of several different factors on the pulse duration, including pulse front tilt, GDD and the Fourier-transform limit. Then, we further accurately evaluate the performance of the monochromator using the pulse propagation method based on Fourier optics.

### 3.1. Theoretical analysis

While utilizing a single grating for the spectral selection, the resolving power *R* = 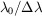

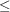
 2.26|*m*|*N* according to the Rayleigh criterion, where *N* is the number of lines within the full width at half-maximum (FWHM) of the intensity distribution of the footprint for a Gaussian source (Nicolas & Cocco, 2022 ▸). The single-grating monochromator will induce a pulse front tilt at the output according to the **K** matrix of the VLS grating, which results in the pulse duration broadening that can be expressed as (Poletto *et al.*, 2018 ▸; Nicolas & Cocco, 2022 ▸; Hu *et al.*, 2023*a* ▸) 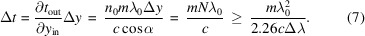
Obviously, the pulse stretching is directly proportional to the number of grooves in an illuminated area on the grating, and the minimum value of the pulse front tilt is quite close to the Fourier-transform limit.

In the design of a single-grating monochromator, pulse stretching caused by front tilt will not significantly exceed the Fourier-transform limit, if ensuring that the number of illuminated grooves times the diffraction order equals the actual resolving power. However, it is not easy to minimize the illuminated area on the grating, due to the significant changes in the light source’s divergence angle and the angle of incidence at different wavelengths, and considering that the incidence angle changes significantly with wavelength. As mentioned by Frassetto *et al.* (2011 ▸) and Nicolas & Cocco (2022 ▸), there are some optimization strategies such as using a slit for clipping the beam or utilizing a bendable elliptical mirror to adjust the beam size adaptively. However, these methods will be accompanied by additional problems, such as a significant reduction of the resolving power or photon flux.

A double-grating monochromator, also called TDCM, has been proposed to reduce the pulse stretching caused by the pulse front tilt (Ito *et al.*, 2010 ▸). According to previous work by Hu *et al.* (2023*b* ▸), the **K** matrix of the double-grating monochromator is given by 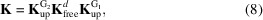
where 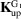, 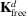 and 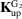 are the **K** matrix of the first VLS plane grating, free space with distance *d*, and the second VLS plane grating, respectively. Using equation (8), the pulse stretching caused by the pulse front tilt can be expressed as 
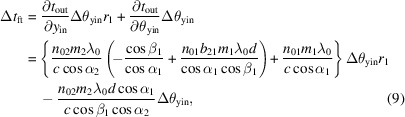
where *r*_1_ is the distance between the source and the first VLS grating, and Δθ_yin_ is the divergence of the incident pulse on the first grating. *n*_01_ and *n*_02_ represent the central groove density of the first and the second VLS plane grating. *m*_1_ and *m*_2_ are the diffraction order of the two gratings, respectively. α_1_, α_2_ and β_1_, β_2_ are the incidence angles and diffraction angles of the two gratings, respectively. *b*_21_ is the VLS parameter of the first grating.

To minimize the pulse stretching caused by the pulse front tilt, it is only necessary to satisfy equation (9) being equal to zero. We can employ the following relationship, 
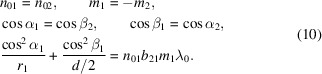
According to equation (10), the design of the TDCM should fulfill the following conditions. (1) The central groove density of the two gratings is identical. The other parameters, such as the law for groove space variation, should also keep identical for symmetry of configuration, although this is not a stringent requirement. (2) The two gratings are mounted symmetrically, which means that the diffraction angle of the first grating is equal to the incidence angle of the second grating. Additionally, the two gratings are used in opposite diffraction orders, faced on the same side and placed at the same distance from the slit, such that the same number of grooves are illuminated on each.

In such an optimized configuration, the second grating provides perfect compensation for the duration broadening caused by the pulse front tilt introduced by the first grating, if the wavefront distortion caused by aberrations and profile errors is not considered. However, this configuration introduces the second effect known as GDD (Yang *et al.*, 2023 ▸). The pulse stretching caused by GDD can be written as 
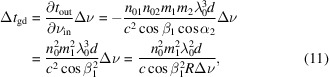
where Δν is the frequency bandwidth after the second grating.

It is evident that the pulse stretching caused by GDD varies linearly with the frequency bandwidth and it is always positive. As the resolving power increases, the pulse stretching decreases significantly, indicating that the pulse stretching caused by GDD can be controlled by adjusting the slit width. However, the pulse duration cannot continually decrease with the reduction of frequency bandwidth. This is because the diffraction effect of the slit breaks the symmetry of the system, causing the area illuminated on the second grating to be larger than that on the first grating. In the design of the TDCM, we should compare the pulse stretching caused by GDD and the Fourier-transform limit and try to find a balance.

### 3.2. Optical layout

**Figure 2:**
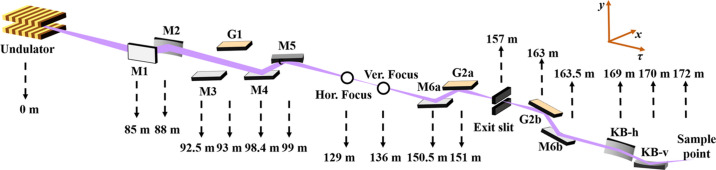
The preliminary optical layout of the ARPES branchline of the FEL-4 beamline.

The FEL-4 beamline operates in SASE mode or EEHG with a wavelength range of 5–30 nm. FEL-4 beamline can deliver XFEL pulses from undulators to four different branch­lines, including the ARPES branchline. As shown in Fig. 2 ▸, we present the preliminary optical layout of the ARPES branchline. The plane offset mirror M1 is placed 85 m downstream of the source. The elliptical mirror M2 horizontally focuses the XFEL beam at a distance of 129 m from the source point. M5c focuses the XFEL beam and generates a vertical focus at 136 m from the source point. The TDCM, used for spectral selection and pulse duration preservation, is composed of two plane mirrors (M6a and M6b) and two blazed VLS gratings (G1a and G1b). The XFEL pulse is finally focused onto the sample by Kirkpatrick–Baez (KB) mirrors (Kirkpatrick & Baez, 1948 ▸).

The main optics specifications of the ARPES branchline are shown in Table 1 ▸. Here, **θ**_in_ represents the grazing incidence angle of the optical elements, and **R** is the radius of curvature.

### 3.3. The temporal response

**Figure 3:**
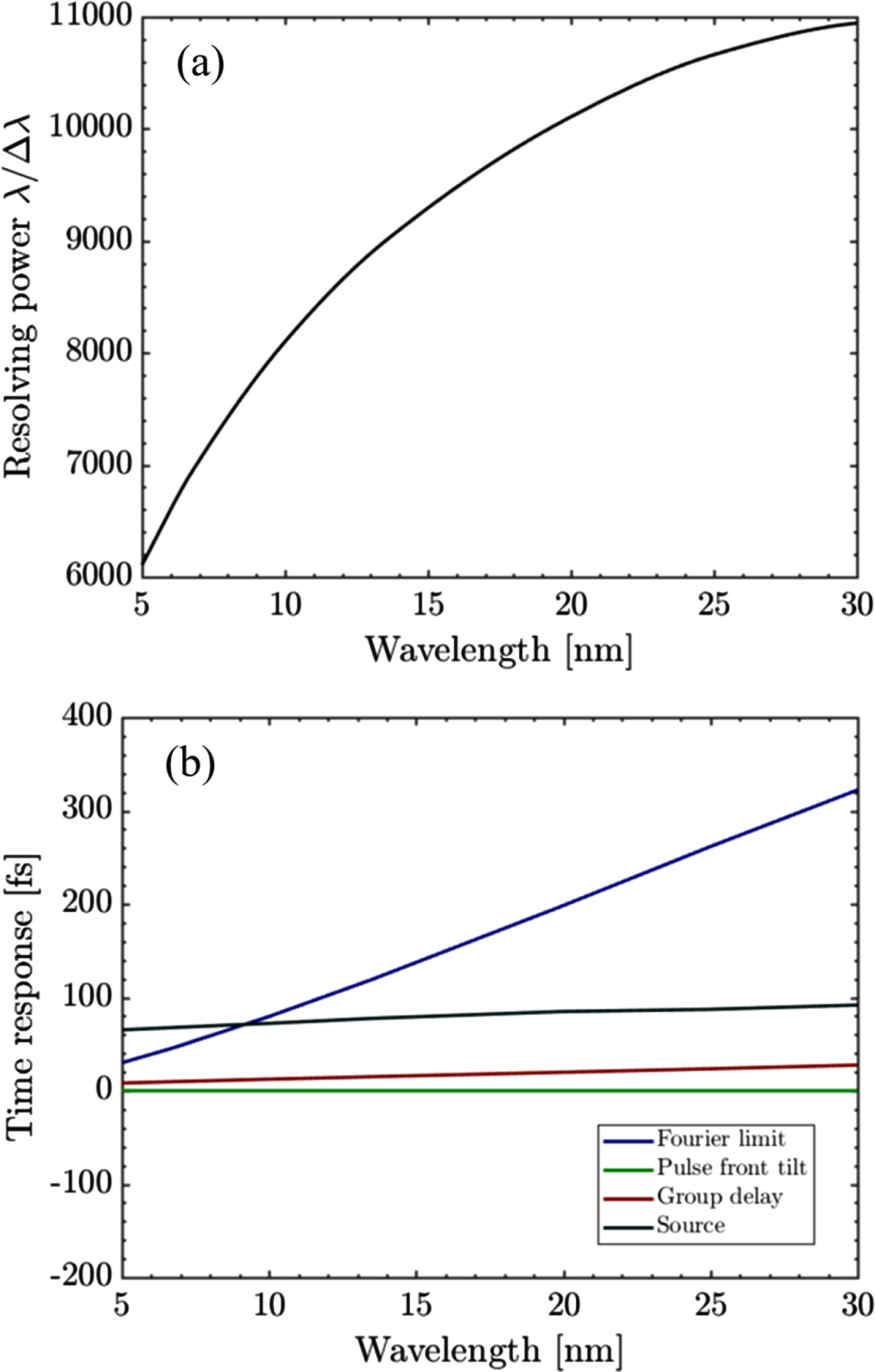
Resolving power and temporal response of the double-grating monochromator. (*a*) The resolving power on a 30 µm slit. (*b*) The temporal response resulting from the pulse front tilt (green), the GDD (brown) and the Fourier-transform limit (blue) at the output of the double-grating monochromator. The original pulse duration at the source is indicated in black.

Considering the requirements to maintain the resolution above 3000, the grating length below than 100 mm, and the time–bandwidth product below 4000 meV fs, the central groove density *n*_0_ and the VLS parameter *b*_2_ of both gratings are set to 600 mm^−1^ and 6.992588 × 10^−4^ mm^−1^, respectively. The two gratings are operated at the +1 and −1 orders, respectively. Fig. 3 ▸(*a*) shows that the resolving power on a 30 µm slit is better than 4000 within the full spectral range, which is sufficient to meet the experimental requirements of the ARPES branchline. The slope errors for both pre-mirrors and gratings are set to 300 nrad. The efficiency of a single blazed grating with carbon coating is expected to be 30–55% within the wavelength range 5–30 nm. The blaze angle that maximizes the grating efficiency is 1.6.

According to the 6-D **K** matrix of the optical system of the ARPES branchline, the temporal response resulting from the pulse front tilt, the GDD and the Fourier-transform limit at the output of the double-grating monochromator are shown in Fig. 3 ▸(*b*). The result indicates that the Fourier-transform limit is the dominating factor affecting the pulse duration, while the impact of GDD and pulse front tilt can be neglected.

The results based on geometric optics, whether **K** matrix or ray tracing, can only be used for a preliminary and approximate estimate of the monochromator’s performances. Therefore, we need to further employ pulse propagation methods based on Fourier optics to obtain a realistic simulation.

### 3.4. Start-to-end simulation

To more accurately assess the characteristics of the FEL pulse after passing through the TDCM, we conduct a start-to-end simulation evaluation of the beamline system. We first produce the 3-D SASE pulses at saturation using *Genesis 1.3* (Reiche, 1999 ▸), and then employ *FURION* to simulate the SASE pulse passing through the beamline system. The FEL simulation parameters are summarized in Table 2 ▸.

**Figure 4:**
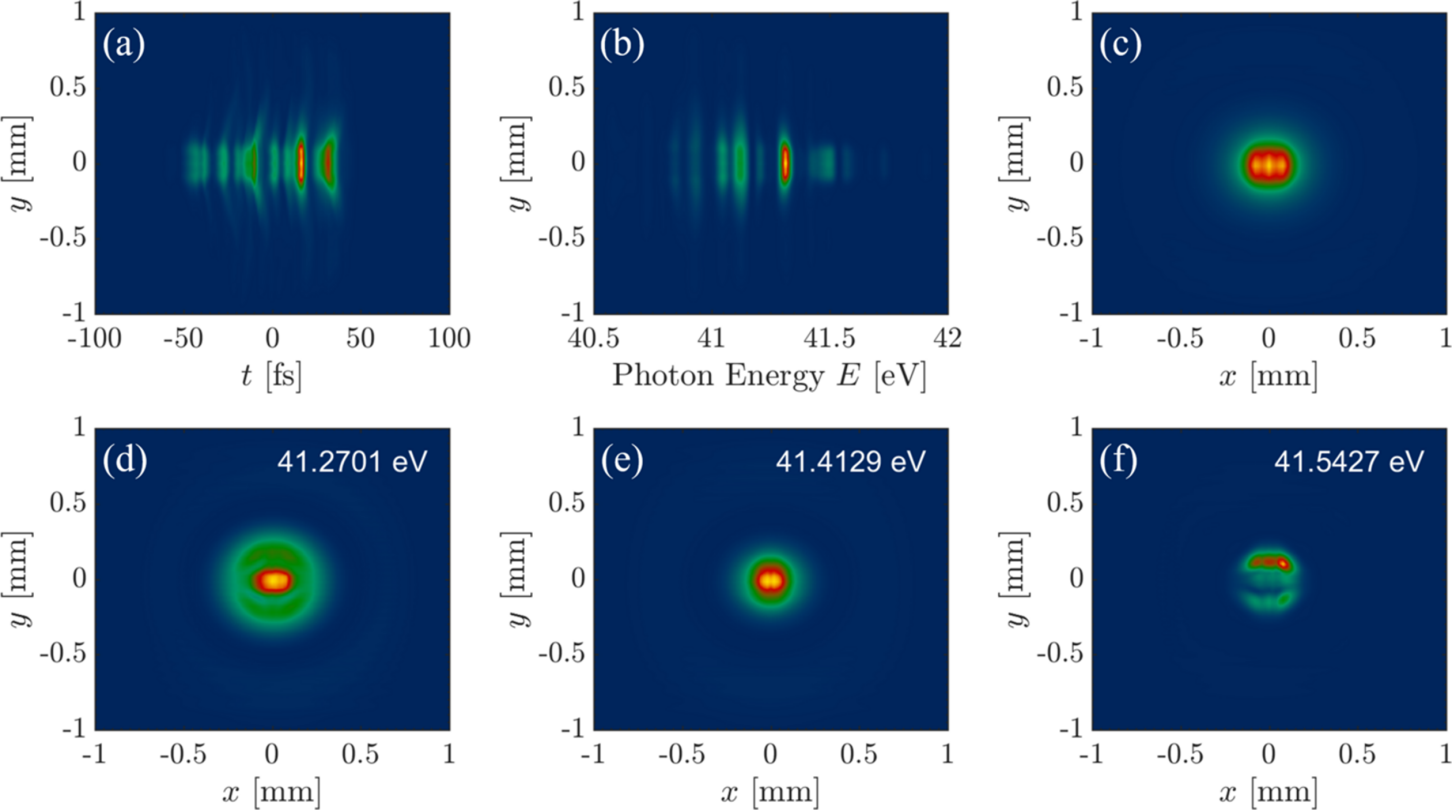
Properties of the 3-D XFEL source. (*a*) The transverse–longitudinal (*y*, *t*) distribution of the pulse. (*b*) The transverse–spectral (*y*, *E*) distribution of the pulse. (*c*) The transverse intensity distribution (*x*, *y*) of the pulse. (*d*–*f*) Transverse distributions of the XFEL pulse at different photon energies.

Fig. 4 ▸(*a*) illustrates the transverse–longitudinal (*y*, *t*) distribution of the pulse and we can observe that the SASE pulse has multiple longitudinal modes. Fig. 4 ▸(*b*) shows the transverse–spectral (*y*, *E*) distribution of the pulse and multiple spikes can be observed in the frequency (photon energy) domain. Fig. 4 ▸(*c*) represents the transverse intensity distribution (*x*, *y*) of the pulse, which is close to a Gaussian distribution. Figs. 4 ▸(*d*), 4(*e*), 4(*f*) represent the transverse distributions of the XFEL pulse at different photon energies. The transverse distribution varies significantly across different photon energies, indicating non-negligible coupling in the transverse and longitudinal directions, which is the primary reason for the partial spatial coherence properties of the pulse. Therefore, the simulation results cannot be simulated only using a single wavefront (*i.e.* coherent light) for propagation. We can utilize the *FURION* model to calculate the propagation of 3-D pulses considering the different frequency components, *i.e.* spatio-temporal coupling of the pulses.

**Figure 5:**
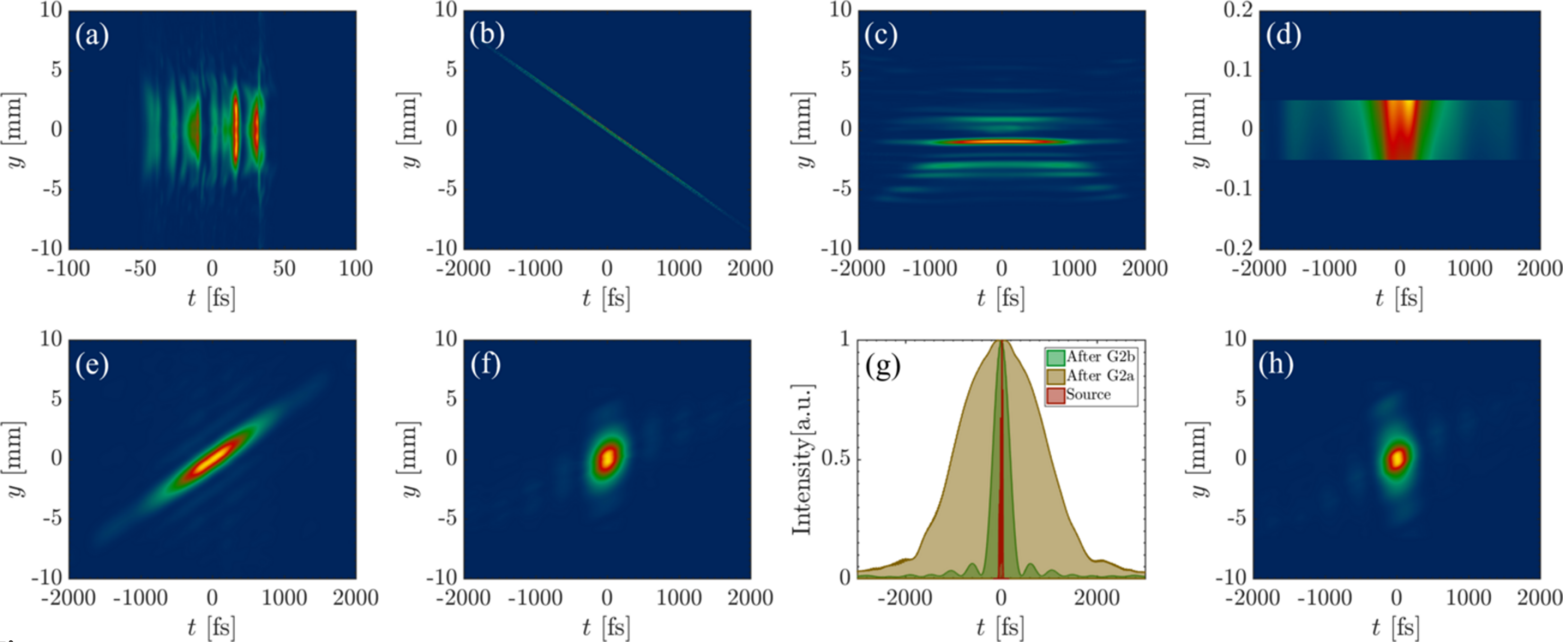
Transverse–longitudinal (*y*, *t*) distribution of the pulse in the ARPES branchline at different locations. (*a*) Before G2a. (*b*) After G2a. (*c*) Before the exit slit. (*d*) After the exit slit. (*e*) Before G2b. (*f*) After G2b. (*g*) The variation of the pulse duration at different positions. (*h*) After G2b by slightly adjusting the incident angle of G2b.

Fig. 5 ▸ presents the numerical simulation results of the XFEL pulses from the source to the ARPES endstation at 30 nm using the double-grating monochromator. Figs. 5 ▸(*a*), 5(*b*), 5(*c*), 5(*d*), 5(*e*) and 5(*f*), respectively, show the projected intensity distribution of the XFEL pulse in (*y*, *t*) space before the first grating G2a, after G2a, before the exit slit, after the exit slit, before the second grating G2b and after G2b. The transverse distribution of the 3-D pulse undergoes modification as it travels from the light source to the location just before G2a, while the temporal duration remains essentially unchanged, due to the absence of dispersive elements in the propagation path. While the pulse traverses the grating G2a, a significant shearing effect is observed in the (*y*, *t*) space, which consequently leads to substantial stretching of the pulse. Dispersion of the pulse occurs at the exit slit, with the XFEL pulse’s spectrum being exhibited in the *y*-dimension, as shown in Fig. 5 ▸(*c*). After passing through a 100 µm slit, the emitted pulse becomes monochromatized with energy bandwidth of 9.44 meV, as shown in Fig. 5 ▸(*d*). While the pulse passes through G2b, Fig. 5 ▸(*e*) shows an opposite pulse shearing effect relative to G2a, thereby compensating for pulse stretching caused by G2a. The monochromatization by the slit, which limits the pulse transversely, disrupts the symmetry of the pulse propagation through the double-grating monochromator, thereby complete pulse compensation cannot be achieved. Due to the shearing effect of G2b and spatio-temporal coupling of the pulse, the diffraction caused by transverse restriction of the slit appears in the temporal dimension, thereby affecting the pulse duration. This is fundamentally based on the fact that it is impossible to maintain the same temporal duration while reducing the energy bandwidth according to the Fourier-transform limit. Fig. 5 ▸(*g*) compares the input FEL pulse duration with the pulse duration after passing through G2a and G2b. In addition, we can observe that there is a slight residual pulse front tilt after G2b in Fig. 5 ▸(*f*), which is primarily caused by the difference between the footprints on the two gratings. The residual tilt can be compensated by slightly adjusting the incident angle of G2b, as shown in Fig. 5 ▸(*h*). Consequently, the pulse duration can be reduced from 380.4 fs to 360.0 fs.

The spectral purity after the double-grating monochromator can be adjusted by altering the size of the exit slit, as shown in Table 3 ▸.

We can observe that the time–bandwidth product can achieve 3400 meV fs at 30 nm and changes slightly with the variation of the slit size. The time–bandwidth product of the Fourier-transform limit is calculated to be 1825 meV fs. This discrepancy primarily results from the fact that the value of the Fourier-transform limit is calculated for an ideal Gaussian pulse, while the spectral distribution is closer to a rectangular distribution due to the constraint by the slit. The time–bandwidth product for an ideal rectangular distribution is about 3664 meV fs, which is approximately twice that of an ideal Gaussian distribution. It can also be observed that the size of the exit slit is the primary factor limiting the pulse duration for a Fourier-transform-limited pulse, due to the time–bandwidth product being close to constant after monochromatization. The pulse duration and energy bandwidth after the monochromator with 50 µm slit size are shown in Table 4 ▸. It shows that the time–bandwidth product is always less than 4000 meV fs and the energy resolution is higher than 5000 within the wavelength range from 5 to 30 nm, which is sufficient to meet the experimental requirements of the ARPES experimental station.

## 4. Conclusions

The preliminary design and expected performance of the ARPES branchline of S^3^FEL have been presented. The beamline delivers 5–30 nm XFEL pulses from undulators to endstations and utilizes a TDCM composed of two VLS gratings in a symmetric layout for spectral selection and pulse duration preservation. As shown in the paper, simulations of the spectral resolution and temporal response are conducted using Fourier optics, which demonstrate a resolving power higher than 5000 and achieve a time–bandwidth product of less than 4000 meV fs. The simulation results based on Fourier optics also demonstrate the impact of the specific spectral distribution on the time–bandwidth product, especially when it deviates from a Gaussian distribution.

## Figures and Tables

**Table 1:** Optics specifications of the ARPES branchline

Optics	Figure	**θ**_in_ (mrad)	**R** (m)	Length (m)
M1	Flat	40	–	0.75
M2	Elliptical cylinder	40	1398.82	0.75
M4	Flat	40	–	0.75
M5c	Elliptical cylinder	40	1347.05	0.75
M6a and M6b	Flat	Scanning	–	0.3
G2a and G2b	Flat VLS	Scanning	–	0.08
KB-h	Bendable	20	–	0.6
KB-v	Bendable	20	–	0.4

**Table 2:** Simulation parameters of *Genesis*

Parameter	Value	Unit
Electron energy	2.5	GeV
Energy spread	0.25	MeV
Peak current	800	A
Photon energy	248 (5 nm), 41.33 (30 nm)	eV
Undulator period	5	cm
Normalized emittance	0.5	mm mrad
Undulator parameter	2.75 (5 nm), 7.45 (30 nm)	–
FEL parameter ρ	0.0013 (5 nm), 0.002 (30 nm)	–
Average beta function	15	m
Transverse beam size (RMS)	30	µm
Bunch length (RMS)	50	fs

**Table 3:** Pulse duration Δ*t* (FWHM), energy bandwidth Δ*E* (FWHM), resolving power and time–bandwidth product Δ*t* Δ*E* for different slit sizes at 30 nm

Slit size	30 µm	50 µm	100 µm	150 µm	200 µm
Δ*t* of source (fs)	92.0	92.0	92.0	92.0	92.0
Δ*t* after G2a (fs)	2080	2080	2080	2080	2080
Δ*t* after G2b (fs)	1290	773	380	253	194
Δ*E* after slit (meV)	3.24	4.94	9.44	13.60	17.55
Resolving power	12757	8367	4357	3039	2355
Δ*t* Δ*E* (meV fs)	4179	3818	3587	3408	3377

**Table 4:** Pulse duration Δ*t* (FWHM), energy bandwidth Δ*E* (FWHM), resolving power and time–bandwidth product Δ*t*Δ*E* with 50 µm slit size at different wavelengths

Wavelength	5 nm	15 nm	30 nm
Δ*t* of source (fs)	60.1	64.2	92.0
Δ*t* after G2a (fs)	195.7	862.7	2080.2
Δ*t* after G2b (fs)	61.3	227.7	773.1
Δ*E* after slit (meV)	49.08	16.15	4.94
Resolving power	5053	5687	8367
Δ*t* Δ*E* (meV fs)	2995	3677	3818
